# Distinct survival strategies in oligotrophic and eutrophic ecotype *Synechococcus*-bacteria co-cultures under iron limitation and warming conditions

**DOI:** 10.1128/mbio.01098-25

**Published:** 2025-06-12

**Authors:** Bowen He, Yu Wang, Min Xu, David A. Hutchins, Fei-Xue Fu, Xiaomin Xia, Ran Duan, Ta-Hui Lin, Nianzhi Jiao, Qiang Zheng

**Affiliations:** 1 State Key Laboratory of Marine Environmental Science, College of Ocean and Earth Sciences, Institute of Marine Microbes and Ecospheres, Xiamen University 554979 , Xiamen, China; 2 Fujian Key Laboratory of Marine Carbon Sequestration, Xiamen University 12466, Xiamen, China; 3 School of Marine Science and Engineering, Hainan University 74629, Haikou, China; 4 Department of Biological Sciences, University of Southern California 118558, Los Angeles, California, USA; 5 Key Laboratory of Functional and Clinical Translational Medicine, Fujian Province University, Xiamen Medical College 5116, Fujian, China,; 6 Department of Basic Medical Science, Xiamen Medical College, Institute of Respiratory Disease 5116, Xiamen, China; Corporación CorpoGen, Bogotá, D.C., Colombia

**Keywords:** iron limitation, warming, *Synechococcus*-bacteria interactions, metatranscriptome, dissolved organic matter, vitamins, siderophores

## Abstract

Phytoplankton-bacteria interactions underpin primary production and nutrient cycling in both oligotrophic and eutrophic aquatic environments, profoundly influencing marine biogeochemical cycles. Despite their importance, how these interactions vary under simultaneous environmental stressors such as warming and iron (Fe) limitation remains largely unexplored, especially across differing ecotypes. Here, we compared the responses of oligotrophic (strain YX04-1) and eutrophic (strain XM-24) ecotype *Synechococcus*-heterotrophic bacteria interactions to concurrent warming and Fe limitation, using the 16S rRNA gene amplicon sequencing alongside metagenomic and metatranscriptomic analyses. Our results revealed that community composition and gene expression in the oceanic *Synechococcus* sp. YX04-1 co-culture were more sensitive to warming, whereas the coastal *Synechococcus* sp. XM-24 co-culture responded more strongly to Fe limitation. The resilience of oligotrophic YX04-1 and its bacterial partners to iron deficiency may result from potential mutualistic triangular dynamics, involving complex carbohydrate decomposition, low-molecular-weight organic substrate transfer, and feedback of public goods. In contrast, the eutrophic XM-24 co-culture experienced intensified competition and opportunistic exploitation of organic resources by dominant mixotrophic bacteria under concurrent warming and Fe limitation conditions. These findings reveal contrasting survival strategies of oligotrophic and eutrophic *Synechococcus*-bacteria co-cultures, highlighting the tighter and mutually beneficial interactions in the oligotrophic co-culture that may assist oligotrophic species in adapting to changing ocean conditions.

**IMPORTANCE:**

Phytoplankton-bacteria interactions serve as a crucial biological network linking primary production and nutrient cycling in marine ecosystems. In the context of global change, the upper ocean inevitably faces increased warming and iron limitation, which will shift primary producer composition toward *Synechococcus* and impact its nutrient exchanges with co-existing bacteria. The changes in this fundamental and widespread microbial interaction may affect the stability of nutrient cycling, yet its universal response under warming and iron limitation remains poorly understood. Our research reveals contrasting responses of oligotrophic and eutrophic *Synechococcus*-bacteria interactions under the same stress, driven by stronger metabolic dependencies in the oligotrophic co-culture but greater individual competitiveness in the eutrophic one. These findings emphasize the importance of cooperative heterotrophic bacteria for host survival and imply a non-uniform co-evolution of *in situ* microbial interactions across different marine ecosystems in the future.

## INTRODUCTION

Phytoplankton and heterotrophic bacteria play critical roles in marine biogeochemical cycles by producing and decomposing organic matter in a dynamically coupled relationship (1–3). *Synechococcus*, for example, contributes ~16.7% of oceanic primary production (4), of which up to 50% is remineralized by heterotrophic bacteria (5). At scales down to the micrometer-level phycosphere, these interactions exhibit tight metabolic interdependence that sustains both photoautotrophic and heterotrophic growth (2). Heterotrophic bacteria rely on diverse algal-derived organic compounds, including polysaccharides, peptides, and monomers such as amino acids (AAs) and monosaccharides (6, 7). In return, they provide inorganic nutrients, vitamins, siderophores, and antioxidants such as superoxide dismutases (SODs), thereby stabilizing the whole microbial community (8, 9). However, these phytoplankton-bacteria interactions are not always mutualistic and may shift depending on resource bioavailability in aquatic environments (10, 11).

Global climate change is causing escalating linked effects on physicochemical parameters in the ocean. Unmitigated global CO_2_ concentrations are projected to increase the surface ocean temperature by 2–3°C over the next century (12). Warming subsequently intensifies surface stratification and inhibits nutrient upwelling, thus magnifying the lack of scarce but vital elements in the ocean, such as iron (Fe) (13). Both warming and Fe limitation are critical factors affecting the growth rate, enzyme activity, physiological process, and nutrient exchange of marine phytoplankton and bacteria (14–16).

Moderate warming enhances the growth rates of pico-phytoplankton and increases the abundance of phycobilisome pigment proteins and other photosynthetic components, particularly in oligotrophic *Synechococcus* strains (17, 18). Additionally, warming promotes the activity of carbonic anhydrase (16), which likely explains the observed carbon fixation stimulation in oceanic *Synechococcus* sp. YX04-1 in our collaborative study focusing on *Synechococcus* (R. Duan, M. Xu, X. P. Bian, C. Y. Kojima, S. W. Hou, Q. Zheng, S. G. John, D. A. Hutchins, and F. X. Fu, submitted for publication). These physiological changes may lead to an increased quantity and bioavailability of phytoplankton-released organic matter (19), promoting carbon flux and shifting bacterial community composition toward organic matter-favoring taxa like *Bacteroidetes* (14, 15).

In addition to temperature, Fe is another crucial factor influencing photosynthesis and the respiratory electron transport chain (20, 21). It also participates in energy production and vitamin synthesis in both phytoplankton and bacteria (20, 22). Fe deficiency likely leads to organic matter-limited environments, intensifying competition for this trace nutrient between phytoplankton and bacteria in both coastal and offshore regimes (23, 24). Nonetheless, heterotrophic bacteria may form cooperative relationships with phytoplankton under Fe stress, providing siderophores in exchange for dissolved organic matter (25). The extent to which *Synechococcus*-bacteria interactions respond to Fe limitation and/or warming remains uncertain, but any such interaction is likely linked to the *Synechococcus*-derived organic compounds.

Fe limitation combined with warming can alter their individual effects, reducing optimum growth temperatures and increasing cellular Fe use efficiencies (IUEs, the rate of carbon fixation per unit of cellular Fe) of multiple phytoplankton (26, 27). These changes may affect phytoplankton mediated carbon export (28), with significant implications for metabolic exchange with associated bacteria. However, our understanding of the interactive effects of these concurrent stressors on phytoplankton-bacteria interactions remains exceedingly limited. The stress-gradient hypothesis posits that facilitative interactions increase with intensified stress among plants (29), but this perspective has not been fully integrated into studies of marine microbial communities. Previous studies have shown that heterotrophic bacteria provide meaningful assistance to *Synechococcus* and *Prochlorococcus* under conditions of nutrient depletion (30) and long-term darkness (31). Nevertheless, whether this cooperation between cyanobacteria and co-existing bacteria under stress is a widespread phenomenon remains to be confirmed.

*Synechococcus* is an excellent model for exploring responses of marine microbial interactions due to its ecological versatility and close associations with co-existing heterotrophic bacteria across diverse habitats from coastal to open ocean environments (32, 33). Previous studies have revealed ecotype-specific differences of *Synechococcus* in Fe requirements (18, 34), stress sensing and regulatory abilities (35), associated bacterial metabolic strategies (36) and responses to concurrent warming and Fe limitation (18). However, it remains unclear how these stressors impact *Synechococcus*-bacterial interactions, and how their impacts vary across different ecotypes. To address this gap, we investigated interactive transcriptomic feedbacks of oligotrophic and eutrophic *Synechococcus*-bacteria co-cultures under warming and Fe limitation. Two *Synechococcus* isolates from the oligotrophic ocean and eutrophic coastal region of the South China Sea, *Synechococcus* sp. YX04-1 (clade II, oligotrophic) and XM-24 (clade CB5, eutrophic), were cultured along with their naturally associated bacterial communities under varying temperatures (20°C, 24°C, and 27°C) combined with either limiting or replete Fe concentrations. 16S rRNA amplicon, metagenomic, and transcriptomic sequencing were used to examine the associated bacterial community composition, identify key bacteria, and integrate metabolic changes. This study aims to explore (i) the combined effects of warming and Fe limitation on typical oligotrophic and eutrophic *Synechococcus*-bacteria co-cultures and (ii) the divergent responses and adaptive mechanisms of heterotrophic bacteria in different ecotype co-cultures and their feedbacks on *Synechococcus*.

## MATERIALS AND METHODS

### Oligotrophic and eutrophic ecotype *Synechococcus* cultures

Oligotrophic ecotype *Synechococcus* sp. strain YX04-1, belonging to subcluster 5.1A clade II, along with its associated heterotrophic bacteria were isolated from oligotrophic seawater of the South China Sea by Q. Zheng. The eutrophic ecotype *Synechococcus* sp. strain XM-24 classified into subcluster 5.2 clade CB5 and its co-existing heterotrophic bacteria were collected from eutrophic coastal seawater near Xiamen Island (37).

### Experimental setup

Triplicate cultures (500 mL) of each *Synechococcus* strain were grown in sterile Aquil medium made with trace metal clean artificial seawater (38). Cultures were grown under a 12:12 dark/light cycle using cool-white fluorescent light at ~30 µmol quanta m^−2^ s^−1^. Based on the optimum growth temperature of the cultures (~24–25°C; Fig. S1) and minimum Fe requirement (~1 nM) of marine cyanobacteria (39), three temperature levels—20°C, 24°C, and 27°C (T20, T24, and T27)—and two Fe concentration levels—2 nM (LFe) and 250 nM (HFe)—were established to simulate a range of warming and Fe availability conditions. All treatments were diluted semi-continuously every other day with sterilized Aquil synthetic ocean seawater to maintain initial cell abundance and sustain the mid-exponential growth phase for at least 2 months (12 generations or more). During dilution, a final concentration of 2 nM Fe was added directly to the LFe cultures.

To ensure minimal Fe contamination, macronutrient solutions were purified by filtration through Chelex 100 resin columns (Bio-Rad Laboratories, Hercules, CA, USA). Reagent additions to the culture medium were performed using sterile pipette tips that had been pre-treated with 10% trace metal clean HCl and rinsed with sterile deionized water. All laboratory glassware underwent a rigorous cleaning process, including overnight immersion in 1% Citranox detergent, followed by multiple rinses with deionized water, a 7 day acid treatment in 10% HCl, and final sterilization using microwave irradiation. DNA and RNA samples were collected from 300 mL cultures, flash-frozen, and stored in liquid nitrogen for subsequent extraction and sequencing.

### DNA-sequencing and 16S rRNA gene amplification

DNA extraction used DNA Miniprep Kit (ZymoBIOMICS) following the manuals. DNA of each culture was first amplified in the V4–V5 hypervariable region of the 16S rRNA genes (515F [5′-GTGYCAGCMGCCGCGGTAA-3′] and 926R [5′-CCGYCAATTYMTTTRAGTTT-3′]) (40, 41). Sequence libraries were constructed using the ALFA-SEQ DNA Library Prep Kit following the manuals. Amplicon sequencing was conducted on the Illumina MiSeq (Illumina, San Diego, CA, USA) platform using PE250 sequencing. Raw sequences were trimmed to 230 bp, removing primer and low-quality sequences (Phred score < 37) using QIIME2 pipeline v2020.2 with default parameters. Remaining sequences were denoised and clustered into amplicon sequence variants (ASVs) using the QIIME2-Deblur pipeline (42).

### Metagenomic analysis and functional profiling

Quality-approved DNA samples were subjected to library preparation using the ALFA-SEQ DNA Library PrepKit following the instructions. Amplified libraries were sequenced on the Illumina platform using PE150 sequencing. Raw sequencing reads were quality-controlled using the read_qc module from metaWRAP v1.3.2 and then assembled by MEGAHIT v1.1.3 with default parameters (43). Medium- and high-quality bins (completeness > 80% and contamination < 10%) were recovered using the metaWRAP binning pipeline (44). Taxonomic information of bins were annotated using GTDB-Tk v2.4.0 (45).16S rRNA gene sequences were extracted from recovered bins using barrnap v0.9 (https://github.com/tseemann/barrnap). Genome abundance was represented by the corresponding ASV abundance based on taxonomic information or 16S rRNA alignment using BLAST v2.11.0 (coverage > 85, identity > 97, and *e*-value < 10^−20^) (46, 47).

Open reading frames were identified by the Prodigal v2.6.3 (48) and then annotated using PROKKA v1.11 (49) against the UniProt database (2021_03). Carbohydrate-active enzyme (CAZyme) and substrate annotation (Table S1) were performed using the dbCAN3-sub database (v12) (50) with the 10^−5^ *e*-value cutoff. Putative target CAZyme-rich gene clusters and polysaccharide utilization loci (PULs) were predicted manually based on CAZyme patterns, *SusCD* genes, and NCBI nr database annotations. Membrane transport genes, along with their predicted substrates and substrate categories (Table S2), were annotated based on the Transporter Classification Database (2016) (51) through DIAMOND v2.0.6, using sensitive mode with an *e*-value cutoff of 10^−5^ to select the best-scoring hits. Siderophore-related genes (Table S3) were examined by FeGenie v1.0 (52) and previous studies (53–55). Genes involved in SOD and vitamin-related pathways (Table S3) were further identified based on KO numbers or gene names. The presence of pathways in bins was identified via KEGG-decoder v1.0.10 (56) or inspected manually, those with over 75% completion were deemed functional.

### RNA-sequencing and metatranscriptomic analysis

RNA extraction was performed separately for each replicate culture using the Direct-zol RNA Miniprep Kit (ZymoBIOMICS). Whole mRNAseq libraries were generated using NEB Next Ultra Nondirectional RNA Library Prep Kit for Illumina following the manufacturer’s recommendations. Libraries were sequenced on an Illumina Novaseq6000 platform using PE150 sequencing. Raw data were processed with FASTP v0.23.0, filtering out reads with a mean quality score < 20 or length < 75 bp (57). Ribosomal RNAs were removed by Bowtie2 v2.33 with SILVA 138 database (58). Qualified reads were further mapped to binned genomes using HISAT2 v2.2.0 (59) with default parameters. Transcript assembly and quantification were performed using StringTie v2.1.7 with default parameters (60). Differentially expressed genes (DEGs) were identified using gene counts with DESeq2 v1.34.0 (61), with a threshold of |log_2_ fold expression change (log_2_FC)| > 1 and Benjamini and Hochberg adjusted *P* value (*P*.adj) < 0.05 (62). Transcripts per million (TPM) were calculated using transcripts of *Synechococcus* and total binned heterotrophic bacteria in each sample separately to quantify gene expression (63). Genes with count sums equaling 0 were removed from the analysis. KEGG functional enrichment analysis of DEGs was performed using clusterProfiler v4.2.2, with a threshold of *P*.adj < 0.05 (64). Heatmaps and other figures were generated using the pheatmap v1.0.12 and ggplot2 v3.4.1 R packages.

### Statistical analysis

Two-way ANOVA with Tukey’s multiple comparison test was used to compare the statistical significance of changes in aiming pathway expression under LFe and HFe treatments across all temperatures, with *P* < 0.05 indicating significance. To analyze metatranscriptomic profiles (TPM-normalized), we performed principal component analysis (PCA) combined with permutational multivariate ANOVA (PERMANOVA), while microbial community composition was assessed using PERMANOVA alone. Differential relative abundance of each bin was further evaluated with ANCOMBC. All statistical analyses—including two-way ANOVA, ANCOMBC, PCA, and PERMANOVA—were implemented in RStudio using the emmeans v1.10.3, ANCOMBC v2.6.0, and vegan v2.6-10 packages.

## RESULTS

### Genomes recovered from oligotrophic and eutrophic ecotype *Synechococcus* co-cultures

Metagenomic sequencing identified representative bacteria in the *Synechococcus* sp. YX04-1 (oligotrophic) and XM-24 (eutrophic) co-cultures. Seven and five medium/high-quality bins with over 85% completeness and less than 10% contamination were reconstructed from the oligotrophic and eutrophic co-cultures, respectively (Table 1). Taxonomic information showed heterotrophic bacteria were primarily classified into *Alphaproteobacteria*, *Gammaproteobacteria*, and *Flavobacteriia*, except Y-bin1, classified into *Rhodothermia*, and Y-bin6, classified into *Phycisphaerae*.

**TABLE 1:** Characteristics and taxanomic information of recovered genomes from *Synechococcus* co-cultures^*a*^

Ecotype	Bin	Completeness (%)	Contamination (%)	Class	Family/genus/species	Size (Mb)
Oligotrophic	*Bal*.Y-bin1	93.98	0.936	*Rhodothermia*	*Balneola*	3.49
	*Mar*.Y-bin2	99.46	0.071	*Gammaproteobacteria*	*Marinobacter*	4.35
	*Rhi*.Y-bin3	99.18	0.387	*Alphaproteobacteria*	*Rhizobium*	3.57
	*Rhi*.Y-bin4	99.14	2.8	*Alphaproteobacteria*	*Rhizobiaceae*	5.65
	*Mam*.Y-bin5	99.59	0.451	*Alphaproteobacteria*	*Mameliella*	5.46
	*Phy*.Y-bin6	97.72	0	*Phycisphaerae*	*Phycisphaeraceae*	3.10
	*Syn*.YX04-1	99.72	0	*Cyanophyceae*	*Synechococcus* sp. YX04-1	2.35
Eutrophic	*Alt*.X-bin1	89.28	8.711	*Gammaproteobacteria*	*Alteromonas*	3.90
	*Fla*.X-bin2	99.45	0.54	*Flavobacteriia*	*Flavobacteriaceae*	3.54
	*Mav*.X-bin3	99.09	0.627	*Alphaproteobacteria*	*Marivita*	4.50
	*Ery*.X-bin4	98.51	0.251	*Alphaproteobacteria*	*Erythrobacter*	2.35
	*Syn*.XM-24	100	0.271	*Cyanophyceae*	*Synechococcus* sp. XM-24	2.35

^*a*^ Y-bin and X-bin refer to binned heterotrophic bacteria from *Synechococcus* sp. YX04-1 and XM-24 co-cultures, respectively.

### Taxonomic and functional composition of *Synechococcus*-associated bacteria

**Fig 1:**
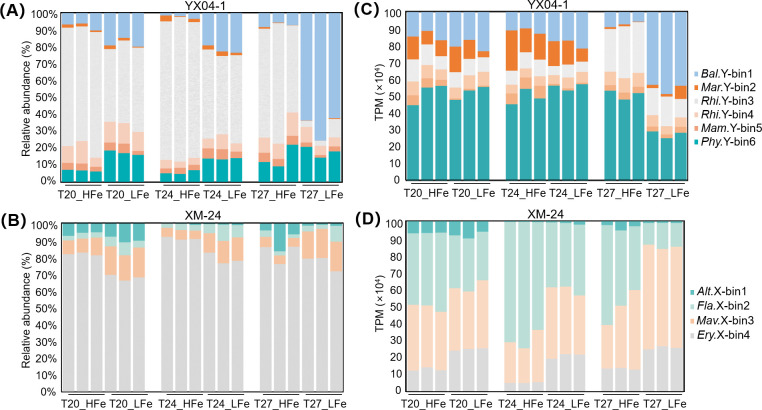
The relative abundance of binned heterotrophic bacteria in *Synechococcus* sp. YX04-1 (**A**) and XM-24 (**B**) co-cultures, as well as transcript expression levels (TPMs) of binned heterotrophic bacteria in *Synechococcus* sp. YX04-1 (**C**) and XM-24 (**D**) co-cultures. Dominant bacteria were shown in gray bars. Alt.X-bin1, *Alteromonas* sp. X-bin1; Bal.Y-bin1, *Balneola* sp. Y-bin1; Ery.X-bin4, *Erythrobacter* sp. X-bin4; Fla.X-bin2, *Flavobacteriaceae* sp. X-bin2; Mam.Y-bin5, *Mameliella* sp. Y-bin5; Mar.Y-bin2, *Marinobacter* sp. Y-bin2; Mav.X-bin3, *Marivita* sp. X-bin3; Phy.Y-bin6, *Phycisphaeraceae* sp. Y-bin6; Rhi.Y-bin3, *Rhizobium* sp. Y-bin3; Rhi.Y-bin4, *Rhizobiaceae* sp. Y-bin4.

Binned bacterial relative abundance (Fig. 1A and B) and gene expression (Fig. 1C and D) were deduced from 16S rRNA sequencing and metatranscriptomics analysis, respectively. Of the total heterotrophic bacterial communities (80 ASVs in oligotrophic and 34 in eutrophic co-cultures), the selected bins represented on average 69% and 77% of sequencing reads across all treatments, respectively (Fig. S2). The dominant heterotrophic bacteria with an average relative abundance >50% of total binned heterotrophic bacteria across treatments were *Rhizobium* sp. Y-bin3 (53%, excluding T27_LFe) in YX04-1 co-culture and *Erythrobacter* sp. X-bin4 (79%) in XM-24 co-culture. The minor bacteria, which averaged less than 15% of total binned heterotrophic bacteria abundance (excluding *Balneola* sp. Y-bin1 in the T27_LFe treatment), contributed over 82% of the total gene expression of binned heterotrophic bacteria.

**Fig 2:**
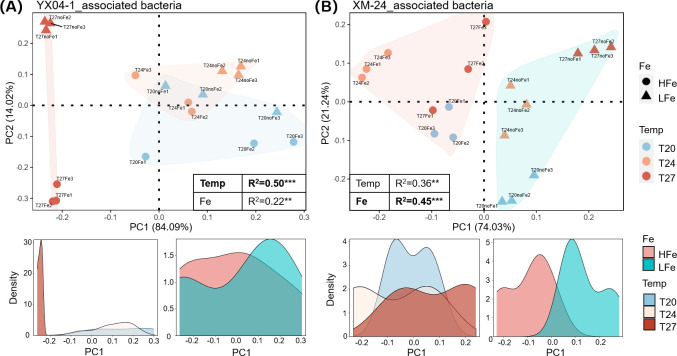
Principal component analysis (PCA) of binned heterotrophic bacterial gene expressions in *Synechococcus* sp. YX04-1 (**A**) and XM-24 (**B**) co-cultures under different treatments. The tables in the PCA plots display PERMANOVA results on the effects of temperature and iron limitation on gene expression variation, with *R*^2^ indicating variance explained and asterisks denoting statistical significance (*P* < 0.01**; *P* < 0.001***). Temperature (Temp) levels are represented by blue (T20), orange (T24), and red (T27), while iron conditions are shown as circles (HFe) and triangles (LFe). Density plots below illustrate the contributions of temperature and iron conditions to gene expression variations, with HFe and LFe treatments in light red and cyan, respectively.

Warming and Fe limitation distinctly affected heterotrophic bacteria in YX04-1 and XM-24 co-cultures. Rising temperatures shifted YX04-1 community composition, with *Balneola* sp. Y-bin1 becoming abundant at T27_LFe (67%) (Fig. 1A). Conversely, Fe deficiency primarily influenced the XM-24 co-culture, slightly decreasing the abundance of dominant *Erythrobacter* sp. X-bin4 while increasing its gene expression by approximately 10%. The relative abundances of certain bins (e.g., *Marinobacter* sp. Y-bin2 and *Alteromonas* sp. X-bin1) declined to around 1% under T27_LFe conditions, which may result from either direct environmental stress or competitive exclusion by dominant bacterial taxa. PCA with PERMANOVA revealed that warming had a significantly stronger impact on bacteria associated with oligotrophic ecotype *Synechococcus* (Fig. 2A; *R*^2^ = 0.50, *P* < 0.001), while Fe limitation affected bacteria associated with eutrophic ecotype *Synechococcus* more profoundly (Fig. 2B; *R*^2^ = 0.45, *P* < 0.001). A similar response pattern was observed in their host *Synechococcus* (Fig. S3), with oligotrophic YX04-1 exhibiting more significantly DEGs under warming compared to eutrophic XM-24 (Duan et al., submitted).

### Metabolic features of *Synechococcus*-associated bacteria

Analysis of DEGs showed that heterotrophs in YX04-1 co-culture exhibited 4,928 upregulated genes (log_2_FC > 1 and *P*.adj < 0.05) and 3,864 downregulated (log_2_FC < −1 and *P*.adj < 0.05) genes, and 1,308 upregulated and 806 downregulated genes in XM-24 associated heterotrophs under varying Fe (LFe vs HFe) and temperature (T20, T24, and T27) conditions. These DEGs were significantly enriched on 64 and 53 KEGG pathways in YX04-1 and XM-24 co-cultures, respectively (Fig. S4; *P*.adj < 0.05).

In the YX04-1 co-culture, most of the enriched pathways in the dominant *Rhizobium* sp. Y-bin3 were downregulated under Fe limitation (Fig. S4A), which were predominantly enriched in central pathways such as the citrate cycle, AA degradation, and carbohydrate metabolism. Lipoic acid metabolism pathway was upregulated in *Rhizobium* sp. Y-bin3, suggesting a potential mechanism to mitigate oxidative stress under low Fe conditions. In contrast, minor bacteria, particularly *Marinobacter* sp. Y-bin2 and *Rhizobiaceae* sp. Y-bin4, enhanced AA and propanoate metabolism, pantothenate and CoA biosynthesis, and quorum sensing pathways. In the XM-24 co-culture, the dominant *Erythrobacter* sp. X-bin4 demonstrated better resilience to environmental pressures, with upregulated pathways related to replication and repair, and cofactor biosynthesis (Fig. S4B).

### Carbohydrate metabolism and PUL repertoires

To evaluate *Synechococcus*-derived carbohydrate utilization, the expression of CAZymes and PULs in heterotrophic bacteria was analyzed across all treatments. At the category level, YX04-1-associated bacteria showed higher total CAZyme expression than XM-24-related bacteria, especially for glycoside hydrolases (GHs), with nearly double expression in the YX04-1 co-culture (RNA-TPM 21,484 vs 11,005; Fig. S5A and B).

**Fig 3:**
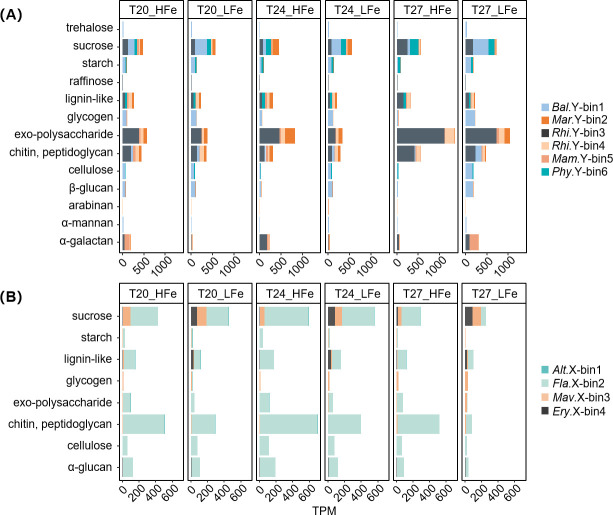
Stacked bar plots display the CAZyme expression for target substrate degradation in binned heterotrophic bacteria from *Synechococcus* sp. YX04-1 (**A**) and XM-24 (**B**) co-cultures under different treatments. Substrates were predicted with dbCAN3-sub. The *x*-axis represents TPM and *y*-axis represents target degraded substrates. Dominant bacteria are shown in black bars. Alt.X-bin1, *Alteromonas* sp. X-bin1; Bal.Y-bin1, *Balneola* sp. Y-bin1; Ery.X-bin4, *Erythrobacter* sp. X-bin4; Fla.X-bin2, *Flavobacteriaceae* sp. X-bin2; Mam.Y-bin5, *Mameliella* sp. Y-bin5; Mar.Y-bin2, *Marinobacter* sp. Y-bin2; Mav.X-bin3, *Marivita* sp. X-bin3; Phy.Y-bin6, *Phycisphaeraceae* sp. Y-bin6; Rhi.Y-bin3, *Rhizobium* sp. Y-bin3; Rhi.Y-bin4, *Rhizobiaceae* sp. Y-bin4.

**Fig 4:**
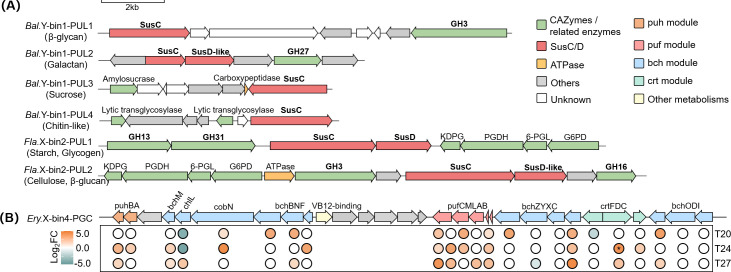
Putative CAZyme-rich gene clusters and PULs involved in targeted substrates utilization in binned heterotrophic bacteria from *Synechococcus* sp. YX04-1 and XM-24 co-cultures (**A**). Photosynthetic gene cluster (PGC) and its expression patterns in the dominant *Erythrobacter* sp. X-bin4 from *Synechococcus* sp. XM-24 co-culture under iron limitation (T20, T24, and T27) (**B**). Asterisks denote significantly changed genes (|log_2_FC| > 1 and *P*.adj < 0.05). Orange and cyan represent upregulated and downregulated genes. 6PGL, 6-phosphogluconolactonase; Bal.Y-bin1, *Balneola* sp. Y-bin1; Ery.X-bin4, *Erythrobacter* sp. X-bin4; Fla.X-bin2, *Flavobacteriaceae* sp. X-bin2; G6PD, glucose-6-phosphate dehydrogenase; KDPG, 2-dehydro-3-deoxy-phosphogluconate aldolase; PGDH, erythrose-4-phosphate dehydrogenase.

When digging deeper into specific substrates, CAZymes were primarily predicted to target α- and β-glucans, peptidoglycan, exo-polysaccharide (EPS), sucrose, and other typical cyanobacteria-derived compounds (65). Generally, most minor bacteria exhibited broader substrate utilization potential than the dominants in both co-cultures (Fig. 3; Fig. S4C and D). Among the minors, *Balneola* sp. Y-bin1 and *Flavobacteriaceae* sp. X-bin2 were identified as active carbohydrate consumers, evidenced by representing 23.89% and 84.67% of total heterotrophic bacterial CAZyme expression in YX04-1 and XM-24 co-cultures, respectively (Fig. 3). Additionally, only these two minors possessed candidate PULs (*susCD* or 1 *susC-*, *susD-like* genes plus at least 1 degradative CAZyme) (Fig. 4A). Notably, although the eutrophic dominant *Erythrobacter* sp. X-bin4 showed weak CAZyme expression, it possessed a complete bacteriochlorophyll-based photosynthetic gene cluster (PGC) comprising two conserved regions, *puhBA-bchMLBNF* and *pufCMLAB-bchZYXC-crtFDC-bchODI* (Fig. 4B). Moreover, most genes involved in the PGC were upregulated in response to Fe limitation across all temperatures (Fig. 4B).

Higher temperatures increased CAZyme expression of YX04-1-associated heterotrophs by 39.66% (T27 vs. average of T20 and T24) (Fig. 3A), while Fe deficiency reduced CAZyme expression in the XM-24 co-culture by 37.81% (average of all temperatures) (Fig. 3B). EPS-degrading CAZymes exhibited peak activity in YX04-1-associated heterotrophs, suggesting EPS served as the primary substrate at high temperature (T27). Other carbohydrates were mainly consumed by *Balneola* sp. Y-bin1, particularly under T27_LFe (contributed 34.88% of total CAZyme expression).

### Transport of diversified low-molecular-weight (LMW) organic substrates

**Fig 5:**
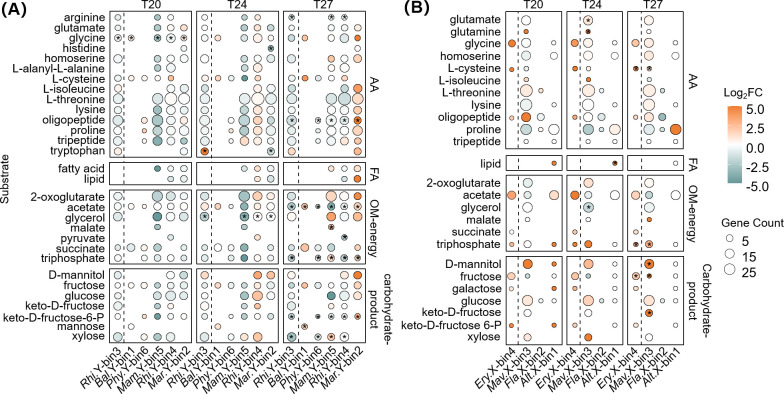
Heatmaps show changes in gene expression patterns of low-molecular-weight substrate transport in binned heterotrophic bacteria from *Synechococcus* sp. YX04-1 (**A**) and XM-24 (**B**) co-cultures under iron limitation at T20, T24, and T27. The left side of the dashed line represents the dominant bacteria in the co-culture. Asterisks denote significantly changed transporters (|log_2_FC| > 1 and *P* < 0.05). Orange and cyan represent upregulated and downregulated transporters, respectively. Bubble size represents the number of genes matching the corresponding function. AA, amino acids; Alt.X-bin1, *Alteromonas* sp. X-bin1; Bal.Y-bin1, *Balneola* sp. Y-bin1; carbohydrate products, secondary products of complex carbohydrates (monosaccharides and derivatives); Ery.X-bin4, *Erythrobacter* sp. X-bin4; FA, fatty acids and lipids; Fla.X-bin2, *Flavobacteriaceae* sp. X-bin2; Mam.Y-bin5, *Mameliella* sp. Y-bin5; Mar.Y-bin2, *Marinobacter* sp. Y-bin2; Mav.X-bin3, *Marivita* sp. X-bin3; OM-energy, energy-generated organic matter; Phy.Y-bin6, *Phycisphaeraceae* sp. Y-bin6; Rhi.Y-bin3: *Rhizobium* sp. Y-bin3; Rhi.Y-bin4, *Rhizobiaceae* sp. Y-bin4.

In addition to complex carbohydrates, the transport of simpler LMW organic substrates was compared between LFe and HFe treatments across all temperatures, including AAs, fatty acids and lipids (FAs), other energy-generating organic matter (OM-energy), as well as mono- or oligo-saccharides and derivatives (carbohydrate products) (Fig. 5). In the YX04-1 co-culture (Fig. 5A), minor bacteria *Rhizobiaceae* sp. Y-bin4 and *Marinobacter* sp. Y-bin2 upregulated transporters of most simple organic substrates (e.g., glycine, oligopeptide, acetate, and d-mannitol) at T24_LFe and T27_LFe, respectively. In the XM-24 co-culture (Fig. 5B), the dominant *Erythrobacter* sp. X-bin4 and minor *Marivita* sp. X-bin3 simultaneously upregulated transporters of simple organic substrates (e.g., l-cysteine, oligopeptide, triphosphate, and fructose) under Fe limitation. Interestingly, the aforementioned bacteria exhibited relatively lower expression of CAZymes targeting complex carbohydrates (e.g., starch, glycogen, β-glucan, cellulose, and α-mannan) but enhanced transport of carbohydrate-derived products, particularly d-mannitol, fructose, glucose, and xylose (log_2_FC > 1). This pattern indicates a clear preference for simple over complex compounds, characteristic of these LMW organic substrate specialists.

### Siderophores, vitamins, and SODs

**Fig 6:**
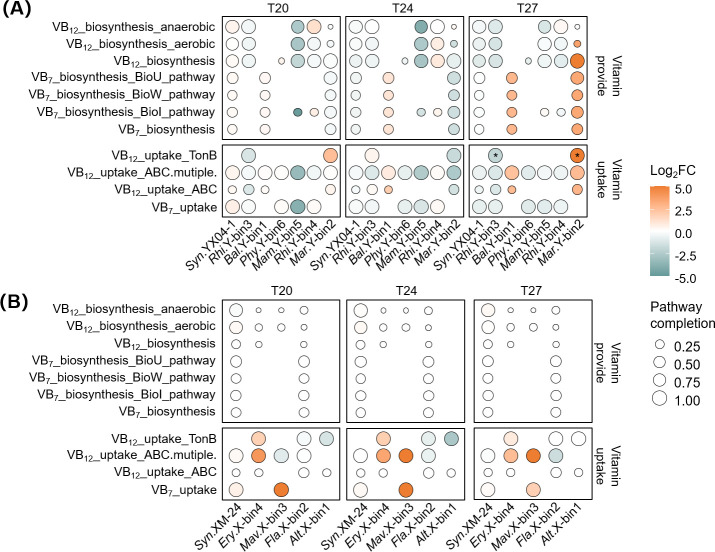
Heatmaps show changes in gene expression patterns of vitamin-related pathways in binned bacteria from *Synechococcus* sp. YX04-1 (**A**) and XM-24 (**B**) co-cultures under iron limitation at T20, T24, and T27. Asterisks denote significantly changed pathways (|log_2_FC| > 1 and *P* < 0.05). Orange and cyan represent upregulated and downregulated pathways, respectively. Bubble size represents pathway completion. Alt.X-bin1, *Alteromonas* sp. X-bin1; Bal.Y-bin1, *Balneola* sp. Y-bin1; Ery.X-bin4, *Erythrobacter* sp. X-bin4; Fla.X-bin2, *Flavobacteriaceae* sp. X-bin2; Mam.Y-bin5, *Mameliella* sp. Y-bin5; Mar.Y-bin2, *Marinobacter* sp. Y-bin2; Mav.X-bin3, *Marivita* sp. X-bin3; Phy.Y-bin6, *Phycisphaeraceae* sp. Y-bin6; Rhi.Y-bin3, *Rhizobium* sp. Y-bin3; Rhi.Y-bin4, *Rhizobiaceae* sp. Y-bin4; Syn.XM-24, *Synechococcus* sp. XM-24; Syn.YX04-1, *Synechococcus* sp. YX04-1; VB_7_: vitamin B_7_; VB_12_: vitamin B_12_.

To elucidate public goods exchanges between *Synechococcus* and co-existing bacteria, we analyzed genes involved in siderophore (Fig. S6) and vitamin (Fig. 6) synthesis and uptake pathways. In the YX04-1 co-culture, the siderophores were primarily produced by *Marinobacter* sp. Y-bin2 (T20 and T27, log_2_FC > 1), meanwhile pyochelin was also supplied by *Rhizobiaceae* sp. Y-bin4 and *Balneola* sp. Y-bin1 (Fig. S6A). Correspondingly, *Synechococcus* sp. YX04-1 and *Balneola* sp. Y-bin1 upregulated pyoverdine and pyochelin uptake-related genes. In the XM-24 co-culture, *Marivita* sp. X-bin3 was the primary potential producer of pyoverdine and pyochelin, while these compounds were largely utilized by the dominant *Erythrobacter* sp. X-bin4 (log_2_FC > 2.5, except for pyoverdine uptake under T27) (Fig. S6B).

The primary potential providers of vitamins B_12_ and B_7_ (VB_12_ and VB_7_) in the YX04-1 co-culture were *Rhizobiaceae* sp. Y-bin4 (VB_12_, all temperatures), *Balneola* sp. Y-bin1 (VB_7_, all temperatures), and *Marinobacter* sp. Y-bin2 (VB_12_ and VB_7_, T27) (Fig. 6A). It is worth noting that *Balneola* sp. Y-bin1 could not produce VB_12_ but increased its uptake-related genes with rising temperatures, potentially from *Rhizobiaceae* sp. Y-bin4 and *Marinobacter* sp. Y-bin2. In contrast, *Synechococcus* sp. XM-24 was the main potential vitamin source in the XM-24 co-culture (Fig. 6B). The synthesized vitamins were mostly used by the dominant *Erythrobacter* sp. X-bin4 (VB_12_, all temperatures) and minor *Marivita* sp. X-bin3 (VB_12_ and VB_7_, T27) (log_2_FC > 1.5).

In addition, SODs were investigated in the two co-cultures (Fig. S7). YX04-1 and associated bacteria harbored more Fe-free SODs than the eutrophic *Synechococcus* and co-existing bacteria. The XM-24 associated bacteria mainly used Fe-SOD, except for *Flavobacteriaceae* sp. X-bin2 (Mn-SOD) and *Erythrobacter* sp. X-bin4 (zinc-SOD-like protein YojM). Moreover, SODs in the YX04-1 co-culture upregulated with rising temperatures, while SODs in the XM-24 co-culture upregulated under Fe limitation regardless of temperature changes.

## DISCUSSION

Synergistic interactions between heterotrophic bacteria and phytoplankton are pivotal for the maintenance of algal growth and the stability of marine nutrient cycling (2). However, variations in these interactions across different ecological types and under varying environmental conditions are understudied. Here, we experimentally co-cultivated oligotrophic and eutrophic *Synechococcus* with heterotrophic bacteria under increasing temperature and Fe limitation conditions for more than 60 days. We found that the oligotrophic co-culture formed mutualistic relationships due to functional complementarity, whereas eutrophic co-culture developed competitive interactions owing to their individual competitiveness. This suggests that co-existing bacteria may collaborate with highly susceptible *Synechococcus* to enhance joint adaptation to challenging environmental conditions.

### Differential responses and active minor heterotrophs in oligo- and eutrophic *Synechococcus* co-cultures

The oligotrophic YX04-1 and eutrophic XM-24 co-cultures shared a similar core microbial community composition (mainly *Alphaproteobacteria* and *Gammaproteobacteria*), but exhibited distinct responses to warming and Fe limitation. In the YX04-1 co-culture, both community composition (PERMANOVA, *R*^2^ = 0.23, *P* < 0.01) and gene expression (PERMANOVA, *R*^2^ = 0.50, *P* < 0.001) of heterotrophic bacteria demonstrated significant sensitivity to rising temperature, leading to a dramatic community shift, with a marked increase in abundance and gene expression of *Balneola* sp. Y-bin1 under T27_LFe condition (Fig. 1A and C). Conversely, the XM-24 co-culture exhibited stronger responses to Fe limitation, showing pronounced fluctuations in community composition (PERMANOVA, *R*^2^ = 0.61, *P* < 0.001) and gene expression (PERMANOVA, *R*^2^ = 0.45, *P* < 0.001), with suppressed abundance and gene expression of the dominant *Erythrobacter* sp. X-bin4 (Fig. 1B and D). Furthermore, we observed a consistent response pattern between *Synechococcus* and its associated bacteria to warming and Fe limitation in both co-cultures (Fig. 2 and Fig. S3), suggesting that the environmental responses of heterotrophic bacteria are associated with their interactions with *Synechococcus* (2, 30).

*Synechococcus* regulates associated bacteria primarily through organic matter release (6). Heterotrophic bacteria with YX04-1 showed higher and more diverse CAZyme expression than those with XM-24 (Fig. 3; Fig. S5A and B), particularly under warming and Fe limitation (Fig. 3A). This suggests enhanced utilization of YX04-1-derived organic substrates, likely facilitated by the inherently lower Fe quotas, adaptive photosynthetic optimization and multiple Fe acquisition strategies of oligotrophic species (18, 34), along with the alleviation of Fe stress under warming conditions (26). In contrast, the eutrophic species tend to conserve organic carbon for energy storage in response to warming and Fe limitation (66, 67), potentially limiting carbohydrate bioavailability in the XM-24 co-culture (Fig. 3B). Increased organic matter exudation favors bacteria enriched with polymer-degrading enzymes (68). In the YX04-1 co-culture, *Balneola* sp. Y-bin1 demonstrated a broad potential for carbohydrate degradation (Fig. S5C) and upregulated CAZyme expression (Fig. 3A), corresponding with its increasing abundance and gene expression under warming and Fe limitation. Therefore, the *Synechococcus*-derived organic matter utilization was likely one of the key factors driving heterotrophic community succession in the oligotrophic YX04-1 co-culture.

Divergent compositional and functional responses to warming and Fe limitation suggest distinct scenarios in the two co-cultures. Despite these differences, minor heterotrophic communities that were less abundant at DNA level exhibited greater transcriptional activity (Fig. 1), demonstrating more diversified and active transcriptomic consumption of *Synechococcus*-derived organic substrates (Fig. 3). In contrast, quantitatively dominant bacteria suffered greater negative effects, resulting in downregulated central carbon metabolisms (YX04-1 co-culture) and upregulated cellular repair pathways (XM-24 co-culture) (Fig. S4). High-abundance communities often endure more pressure from environmental changes (69), while low-abundance bacteria more actively interact with associated host plants (70). These results suggest that the abundance of a microbial group does not necessarily reflect its ecological significance, as low-abundance bacteria can also play crucial roles in shaping phytoplankton-bacteria interactions.

### Metabolic dependencies established potential mutualistic triangular dynamics within the oligotrophic *Synechococcus* and its associated minor bacteria

The oceanic *Synechococcus* sp. YX04-1 demonstrated a higher growth rate under concurrent heat and Fe stress than coastal XM-24 (18). This better growth performance suggests a potentially more mutualistic relationship in the YX04-1 co-culture, consistent with prior evidence that symbiotic associations with heterotrophic bacteria enhance *Synechococcus* growth rates under environmental stress (19). The foundation of a synergetic mode involves host-released organic matter (9). We observed a notable rise in EPS-related CAZyme expression within the YX04-1 co-culture (Fig. 3A), which could be a response to increasing temperature. EPS can serve as a nutritious matrix to attract beneficial bacteria (47) and a signal for increasing bacterial communication (71). Increasing bacterial communication allows bacteria to operate in unison and regulate processes, such as organic matter degradation, carbon allocation, and nutrient acquisition (72). Thus, the increased EPS suggests both a broad selection of surrounding bacteria by YX04-1 and a more intimate interspecies nutrient exchange, potentially involving polysaccharides, AAs, and vitamins (73).

Complex polysaccharides exudated by phytoplankton primarily maintain bacterial growth (6). However, only about half of bacterial taxa have the necessary enzymes and PULs to utilize carbon-rich exopolysaccharides (74). The type-species in the phylum *Rhodothermaeota* have been reported to specialize in degrading polysaccharides (75). Among them, *Balneola* sp. Y-bin1, a species within *Rhodothermaeota*, contained multiple GHs (e.g. GH13, GH3, and GH38) and was the only microbe possessing PULs in the YX04-1 co-culture (Table S1; Fig. 4A). Consequently, based on gene expression patterns, it excelled at degrading various substrates and became the primary consumer of starch, sucrose, α-mannan, and other typical phytoplankton-derived organic substrates, particularly in the T27_LFe treatment (Fig. 3A).

Complex polysaccharide consumers like *Balneola* sp. Y-bin1 act as gatekeepers for algal glycans, breaking them down into hydrolyzed products like mono- and oligosaccharides, which are then utilized by other bacteria that prefer simpler LMW substrates (37, 47). *Rhizobiaceae* sp. Y-bin4 and *Marinobacter* sp. Y-bin2 exhibited lower polysaccharide-degrading gene expression compared to *Balneola* sp. Y-bin1 in the YX04-1 co-culture (Fig. 3A). However, they effectively enhanced expression of ABC and Fru transporters for LMW substrates like D-mannitol, fructose, and glucose (Fig. 5A; Table S2), which are mostly hydrolyzed from polysaccharides such as starch, sucrose, and α-mannan by *Balneola* sp. Y-bin1. These bacteria also upregulated transporters of other LMW compounds crucial for energy production, including AAs, fatty acids, and glycerol (76, 77) (Fig. 5A). This indicates a potentially sequential substrate utilization in the YX04-1 co-culture, whereby complex polysaccharide consumers supplied simpler organic matter and LMW substrate specialists acted as recipients.

Cooperation is usually not a one-sided effort but stems from the exchange of public goods (78), such as siderophores and vitamins. Our findings revealed that *Rhizobiaceae* sp. Y-bin4 and *Marinobacter* sp. Y-bin2, the main recipients of LMW substrates, were also the primary potential producers of siderophores and vitamins (Fig. S6A). Siderophores can serve as crucial Fe sources and mediate microbial interactions under Fe scarcity (79, 80). Under Fe limitation, *Rhizobiaceae* sp. Y-bin4 and *Marinobacter* sp. Y-bin2 upregulated siderophore biosynthesis and export-related genes across all temperatures, potentially providing pyoverdine and pyochelin not only to YX04-1, but also to *Balneola* sp. Y-bin1 (Fig. S6A), which was breaking down the high-molecular weight carbohydrates for them. This suggested a siderophore-mediated cross-feeding strategy to cope with Fe deficiency in the YX04-1 co-culture.

**Fig 7:**
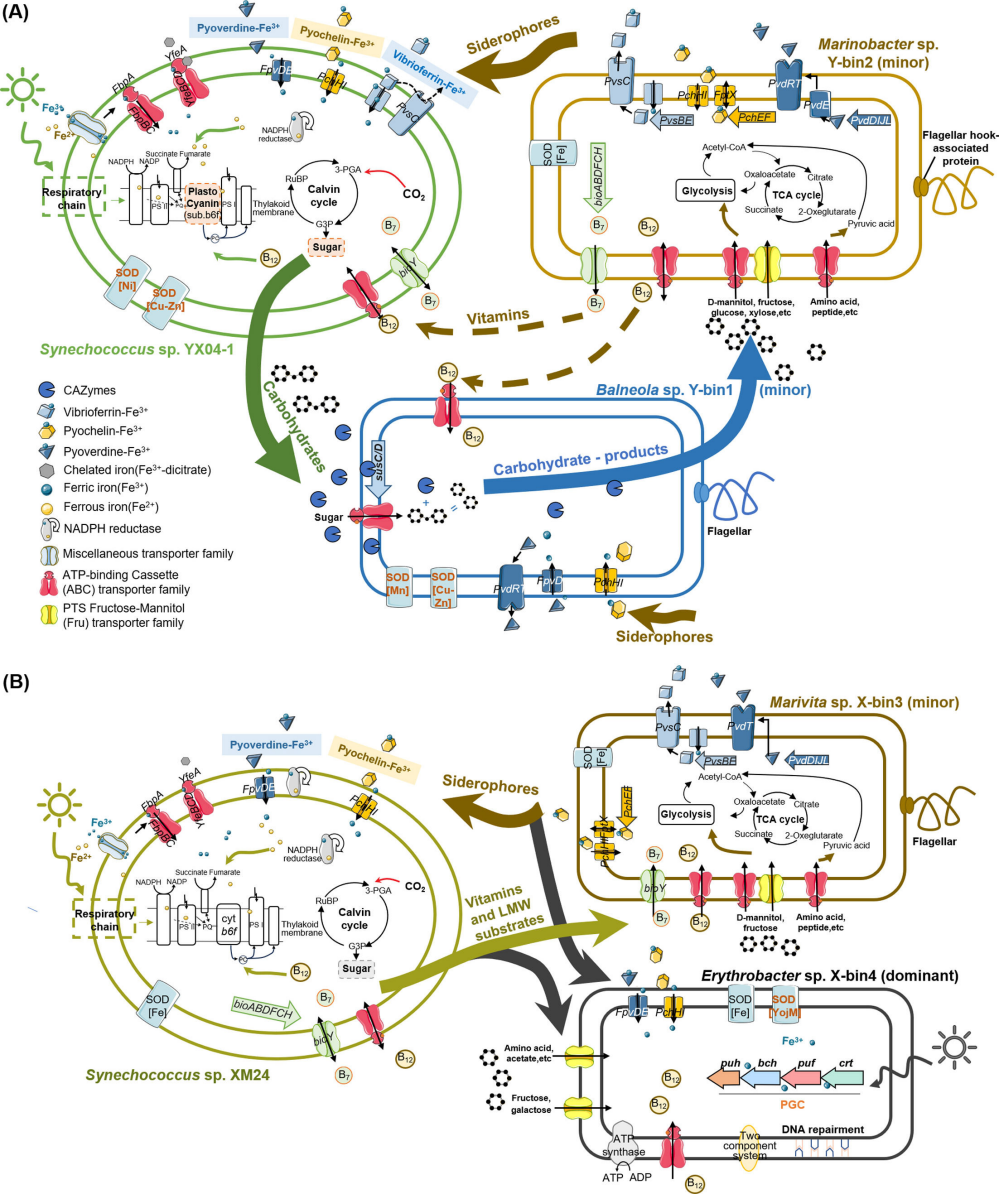
Predicted schematic model of integrated metabolic conversations within *Synechococcus* sp. YX04-1 (**A**) and XM-24 (**B**) co-cultures under warming and iron limitation. The primary nutrient exchanges in *Synechococcus*-bacteria interactions are highlighted by bold colored arrows. *Synechococcus* (*Synechococcus* sp. YX04-1, green; *Synechococcus* sp. XM-24, yellowgreen) released organic substrates produced through photosynthesis into the co-cultures. Polysaccharide consumer (*Balneola* sp. Y-bin1, blue) degraded complex carbohydrates into mono- and oligosaccharides along with their derivatives. Low-molecular-weight (LMW) substrate specialists (*Marinobacter* sp. Y-bin2, yellow; *Marivita* sp. X-bin3, brown) transported simple organic substrates and provided siderophores and vitamins, or mainly siderophores. Competitive dominant bacteria (*Erythrobacter* sp. X-bin4, gray) exhibited photoheterotrophy as well as strong regulatory and repair abilities, exploiting LMW substrates, siderophores and vitamins in competition with other minor bacteria. 3-PGA, 3-phosphoglycerate; Acetyl-CoA, acetyl coenzyme A; B_12_, vitamin B_12_; B_7_, vitamin B_7_; b6f, cytochrome b6f complex; G3P, glyceraldehyde-3-phosphate; PC, plastoquinone; PQ, plastoquinone; PSII, photosystem II; PSI, photosystem I; RuBP, ribulose-1,5-bisphosphate; SOD, superoxide dismutase; TCA cycle, tricarboxylic acid cycle.

Additionally, Fe deficiency negatively impacts the synthesis of vitamins B_7_ and B_12_, which are crucial for photosynthesis, AA, and fatty acid synthesis (81, 82). *Rhizobiaceae* sp. Y-bin4 and *Marinobacter* sp. Y-bin2 upregulated vitamin production-related genes under T24_LFe and T27_LFe conditions, respectively (Fig. 6A). They largely supported the VB_12_ uptake by *Balneola* sp. Y-bin1, the primary complex polysaccharide consumer, which was unable to meet its own vitamin needs. Overall, the potential reciprocal exchange of public goods, polysaccharides and their by-products fostered ecological dependencies among YX04-1, complex polysaccharide consumers and LMW substrate recipients, forming a triangular synergetic scenario that supported diversification and nutrient cycling of the entire community (Fig. 1 and 7A).

### Competition between minor bacteria and dominant aerobic anoxygenic photoheterotrophs in the eutrophic co-culture

In the eutrophic XM-24 co-culture, Fe limitation was the primary stressor among all treatments, potentially resulting in reduced polysaccharide exudation (Fig. 2 and 3B). Transcriptomic data identified *Flavobacteriaceae* sp. X-bin2, the sole carrier of PULs in the XM-24 co-culture, as the primary consumer of polysaccharides (Fig. 3B). Based on PULs and GHs such as GH13, GH3, and GH31 (Fig. 4A; Table S1), *Flavobacteriaceae* sp. X-bin2 could degrade various substrates including sucrose, peptidoglycan, and cellulose. However, the utilization of all substrates was significantly suppressed under T27_LFe (*P* < 0.05), suggesting severely reduced polysaccharide utilization and potential organic substrate scarcity in the XM-24 co-culture under combined heat and Fe stress.

Under low-resource conditions, increased survival capacity can be a crucial adaption to environmental fluctuations (83). *Erythrobacter* sp. X-bin4, unlike the weakened dominant in the YX04-1 co-culture, maintained its abundance dominance in the XM-24 co-culture under adverse conditions (Fig. 1). It significantly upregulated DNA replication and repair pathways (*P.*adj < 0.05) and possessed a unique zinc-dependent SOD-like protein (Fig. S4B and 7B), indicating a strong adaptative ability under stress. Furthermore, *Erythrobacter* sp. X-bin4 uniquely harbored a complete PGC, which was highly upregulated under Fe-limited conditions (Fig. 4B). PGC, a conserved marker for aerobic anoxygenic photoheterotrophic bacteria, enables photoheterotrophy and contributes up to 20% of their total metabolic energy (84). These findings suggested that *Erythrobacter* sp. X-bin4 uniquely integrated phototrophic and heterotrophic capabilities with high-stress resilience, providing significant survival and competitive advantages in adverse environments.

Resource shortages often lead to intense exploitative competition among bacteria (85). LMW organic matter and public goods are widespread and readily available in the environment (86), with fairly rapid turnover rates compared to high-molecular-weight components (87). This makes them particularly susceptible to competition and exploitation. Indeed, our analysis revealed intense competition between the competitively dominant *Erythrobacter* sp. X-bin4 and the minor *Marivita* sp. X-bin3 for AAs, carbohydrate by-products and vitamins under Fe limitation, irrespective of temperature (Fig. 5B and 6B). *Marivita* sp. X-bin3, while reaping benefits, also gave feedback, providing siderophores to both XM-24 and *Erythrobacter* sp. X-bin4 (Fig. S6B). In contrast, *Erythrobacter* sp. X-bin4 acted as a cheater in the co-culture, exploiting VB_12_ and other public goods with fewer contributions to the potential suppliers (Fig. 6B; Fig. S6B). Taken together, the XM-24 co-culture displayed a competitive scenario wherein dominant bacteria outcompeted minor bacteria for LMW substrates and exploited public goods under combined thermal and Fe stress (Fig. 7B). The individual survival needs overshadowed collective benefits, favoring those with superior survival competitiveness.

### Ecological implications for positive support from heterotrophic bacteria

Although laboratory incubations have limited extrapolation to complex natural ecosystems owing to the lack of grazing and other factors, our study still reveals a high-level heterogeneity in oligotrophic and eutrophic *Synechococcus*-bacterial interactions under concurrent warming and Fe limitation (Fig. 7). Only the oligotrophic *Synechococcus*-bacteria interactions support the stress-gradient hypothesis, showing cooperation under environmental stress, while the eutrophic co-culture mainly displayed competition. This discrepancy likely stems from their intrinsic functional traits, such as the lower Fe requirements of oligotrophic species and carbon storage strategies of eutrophic species (18, 67). These intrinsic traits are likely shaped by their different habitats, potentially leading to a non-uniform co-evolution of *Synechococcus*-bacteria interactions across different marine ecosystems in the future.

The positive support from heterotrophic bacteria may profoundly influence the ecological role and value of *Synechococcus* under adverse conditions. Fe deficiency significantly constrain phytoplankton growth and primary production, especially in high-nutrient, low-chlorophyll oceans (13). Small-sized pico-phytoplankton are predicted to be more dominant primary producers in the context of global change due to their high surface-area-to-volume ratio and reduced nutrient demands (88, 89). Our findings suggest that the assistance provided by heterotrophic bacteria may also be critically significant for the adaptation of *Synechococcus* to future warming and Fe-limited oceans. Furthermore, *Synechococcus*-bacteria interactions are strongly associated with carbon export processes in the oligotrophic ocean (90). This indicates a more significant role of cooperative heterotrophic bacteria in enhancing the ecological value of *Synechococcus* in the future marine carbon cycle.

### Conclusion

This study compared oligotrophic and eutrophic ecotype *Synechococcus*-bacteria interactions under concurrent warming and Fe limitation, revealing distinct survival strategies between the two co-cultures. The oligotrophic YX04-1 co-culture established potential mutualistic triangular dynamics, where the *Balneola* strain upregulated CAZyme expression to break down YX04-1-derived polysaccharides (e.g., starch, sucrose, and α-mannan) into LMW substrates (e.g., d-mannitol, fructose, and glucose) for other minor bacteria. In return, these recipients provided siderophores and vitamins. Conversely, the eutrophic XM-24 co-culture reduced polysaccharide utilization under Fe stress, accompanied by competition for other resources (e.g., oligopeptide, triphosphate, fructose, and public goods) between the minor *Marivita* strain and the dominant photoheterotrophic *Erythrobacter* strain. The individual survival needs impeded mutual assistance within the XM-24 community, while the highly susceptible YX04-1 established synergetic dependencies with surrounding bacteria, enhancing its adaptability to adverse conditions. These findings highlight the non-uniform responses of different ecotype *Synechococcus*-bacterial interactions and the critical support provided by heterotrophic bacteria to oligotrophic strains under stress, contributing to enhanced survival potential and ecological value of both *Synechococcus* and its associated microbiome in future oceans.

## Data Availability

The raw 16S rRNA gene sequence, metagenomic sequence, and assembled genomes were deposited in the NCBI database under the BioProject accession no. PRJNA1196138. The raw metatranscriptomic sequence reads were deposited under the BioProject accession no. PRJNA1101000.

## SUPPLEMENTAL MATERIAL

Supplemental figures mbio.01098-25-s0001.docx Fig. S1-S7.

Table S1 mbio.01098-25-s0002.xlsx Carbohydrate-active enzymes and predicted substrates.

Table S2 mbio.01098-25-s0003.xlsx Low-molecular-weight substrate membrane transport genes.

Table S3 mbio.01098-25-s0004.xlsx Siderophore-, vitamin-, and SOD-related genes.

ASM does not own the copyrights to Supplemental Material that may be linked to, or accessed through, an article. The authors have granted ASM a non-exclusive, world-wide license to publish the Supplemental Material files. Please contact the corresponding author directly for reuse.
