# Supplementary material for: Distinct survival strategies in oligotrophic and eutrophic ecotype Synechococcus-bacteria co-cultures under iron limitation and warming conditions
Source: mBio. 2025 Jun 12;16(7):e01098-25. doi: 10.1128/mbio.01098-25 (PMC12239562; doi:10.1128/mbio.01098-25)
Supplement: Supplemental figures — Fig. S1-S7. [file mbio.01098-25-s0001.docx]

**Supplementary Material****s**

**Title: Distinct Survival Strategies in Oligotrophic and Eutrophic ecotype *Synechococcus*-Bacteria co-cultures under Iron Limitation and Warming Conditions**

**Running title: Distinct *Synechococcus*-Bacteria Fe-Warming Responses**

Bowen He^1, 2^, Yu Wang^1, 2^, Min Xu^3^, David A. Hutchins^4^, Fei-Xue Fu^4^, Xiaomin Xia^1, 2^, Ran Duan^4^, Ta-Hui Lin^1, 2, 5, 6^, Nianzhi Jiao^1, 2^, Qiang Zheng^1, 2,#^

**AUTHOR affiliation**

^1^ State Key Laboratory of Marine Environmental Science, College of Ocean and Earth Sciences, Institute of Marine Microbes and Ecospheres, Xiamen University, Xiamen 361102, PR China

^2^ Fujian Key Laboratory of Marine Carbon Sequestration, Xiamen University, Xiang’an Campus, Xiang’an South Road, Xiamen 361102, China

^3^ School of Marine Science and Engineering, Hainan University, Haikou 570228, China

^4^ Department of Biological Sciences, University of Southern California, Los Angeles, USA

^5^ Key Laboratory of Functional and Clinical Translational Medicine, Fujian Province University, Xiamen Medical College, Fujian, China

^6^ Institute of Respiratory Disease, Department of Basic Medical Science, Xiamen Medical College, Xiamen 361023, China

^#^ Corresponding authors: Qiang Zheng

**E-mail address:** zhengqiang@xmu.edu.cn

**Postal address:** State Key Laboratory of Marine Environmental Science, College of Ocean and Earth Sciences, Institute of Marine Microbes and Ecospheres, Xiamen University, Xiamen 361102, PR China

**Supplementary Figures**


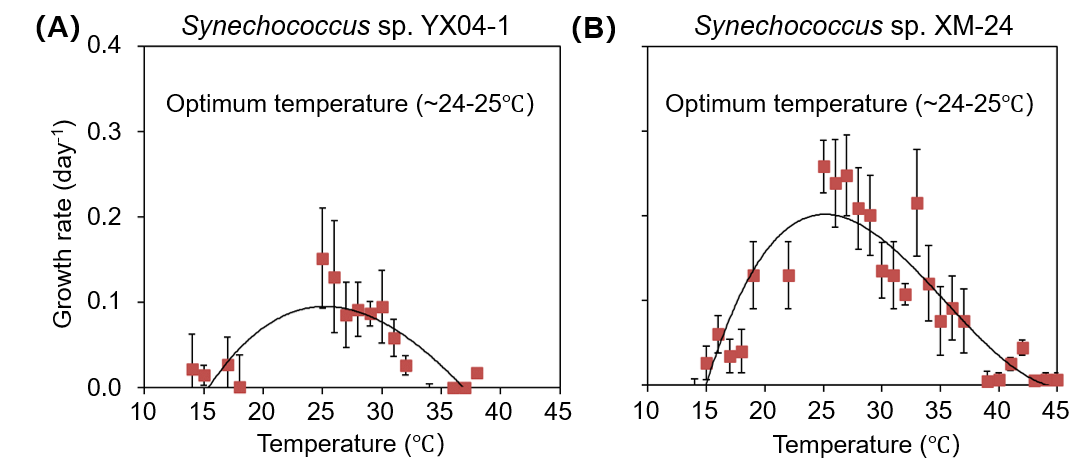


FIG S1 Temperature dependence of *Synechococcus* sp. YX04-1 (A) and XM-24 (B) across temperatures ranging from 14°C to 38°C. The growth rate was calculated from OD_680_ (optical density at 680 nm) values measured by the spectrophotometer (Tecan M200, Männedorf, Switzerland), using the equation: $Growth rate= \frac{\Delta\ln OD680}{\Delta t}$, where *t* is time. Growth rate data were fitted to a quadratic polynomial function using Microsoft Excel.


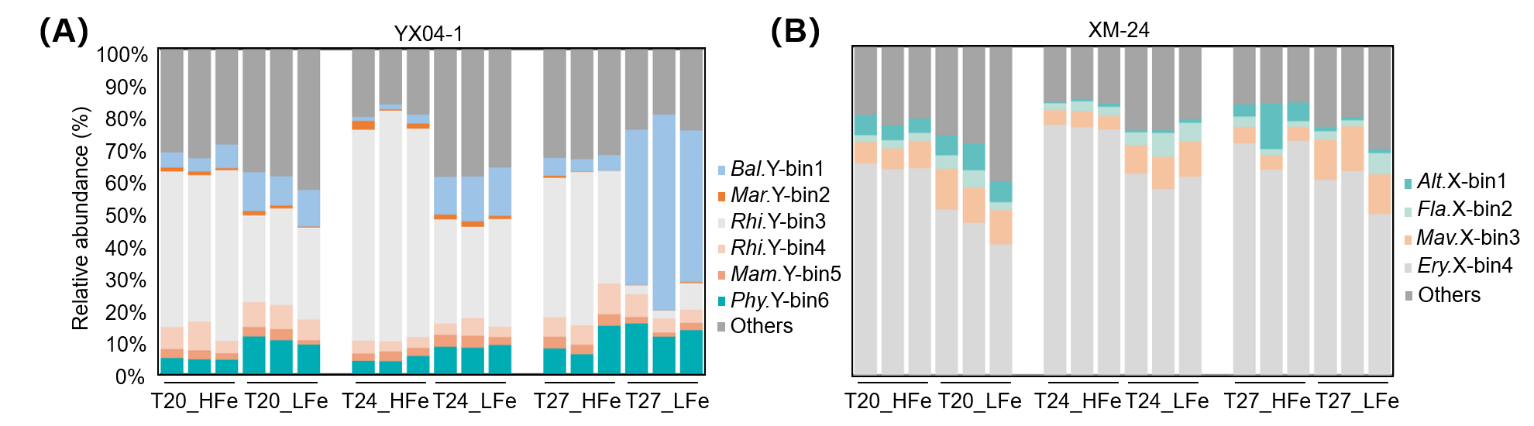


FIG S2 The relative abundance of total heterotrophic bacteria in *Synechococcus* sp. YX04-1 (A) and XM-24 (B) co-cultures. Abbreviations: *Bal.*Y-bin1: *Balneola* sp. Y-bin1; *Mar*.Y-bin2: *Marinobacter* sp. Y-bin2; *Rhi*.Y-bin3: *Rhizobium* sp. Y-bin3; *Rhi*.Y-bin4: *Rhizobiaceae* sp. Y-bin4; *Mam*.Y-bin5: *Mameliella* sp. Y-bin5; *Phy*.Y-bin6: *Phycisphaeraceae* sp. Y-bin6; *Alt*.X-bin1: *Alteromonas* sp. X-bin1; *Fla*.X-bin2: *Flavobacteriaceae* sp. X-bin2; *Mav*.X-bin3: *Marivita* sp. X-bin3; *Ery*.X-bin4: *Erythrobacter* sp. X-bin4; Others: unbinned heterotrophic bacteria.


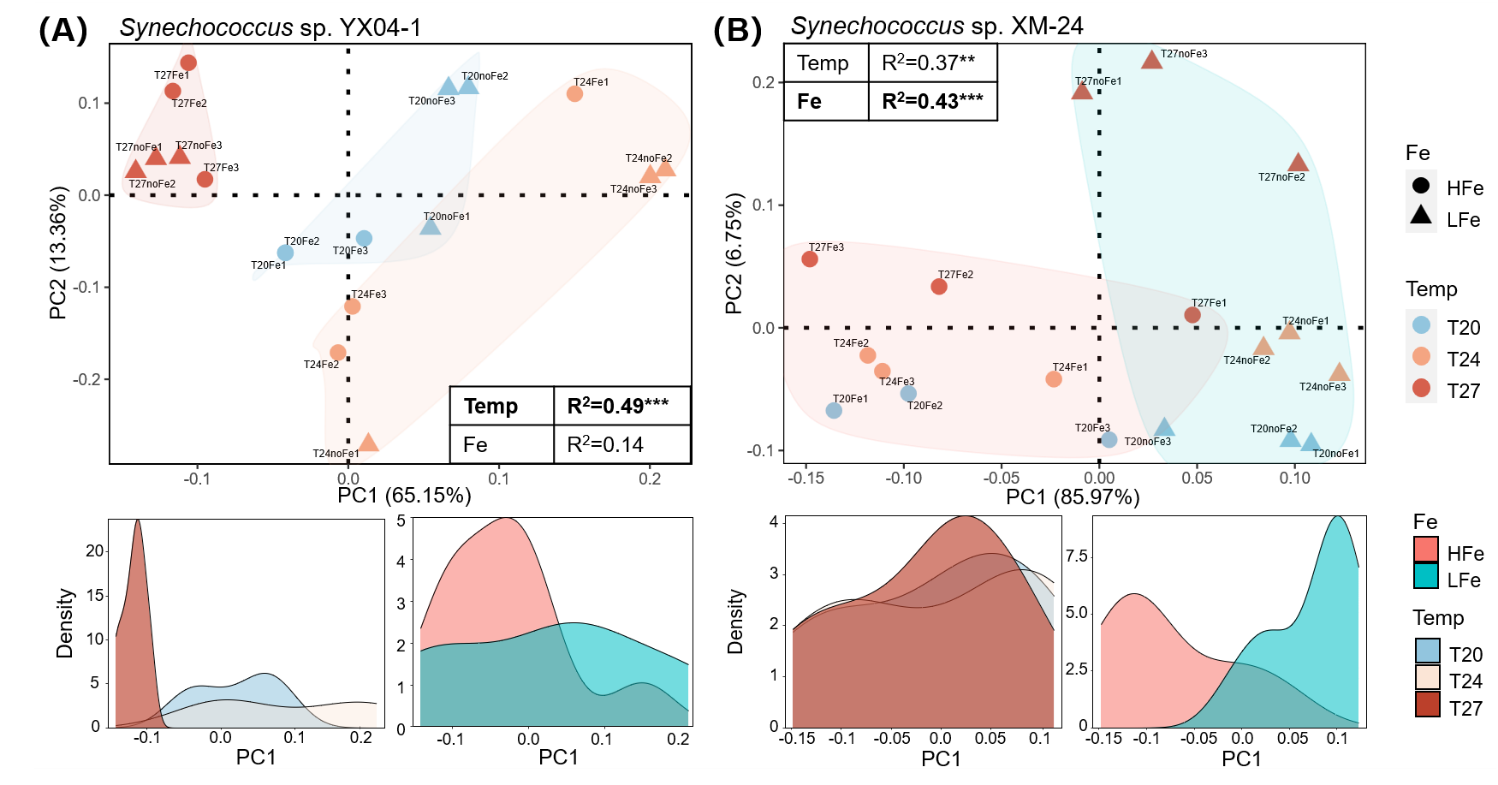


FIG S3 Principal components analysis (PCA) of gene expressions of *Synechococcus* sp. YX04-1 (A) and XM-24 (B) under different treatments. Tables in the PCA plots display PERMANOVA results on the effects of temperature and iron limitation on gene expression variation, with R^2^ indicating variance explained and asterisks denoting statistical significance (*p* < 0.01 **; *p* < 0.001 ***). Temperature (Temp) levels are represented by blue (T20), orange (T24), and red (T27), while iron conditions are shown as circles (HFe) and triangles (LFe). Density plots below illustrate the contributions of temperature and iron conditions to gene expression variations, with HFe and LFe treatments in light red and cyan, respectively.


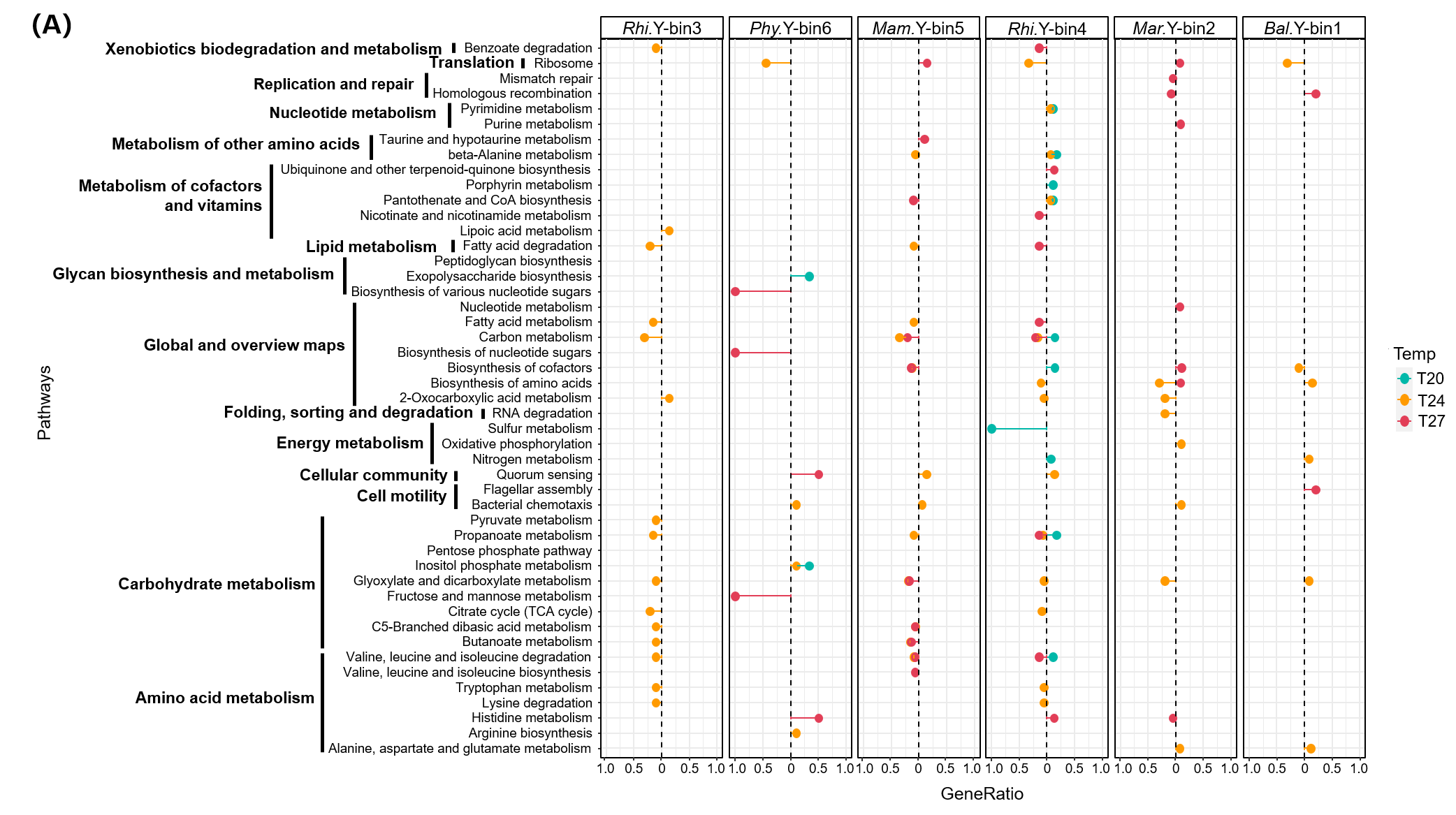


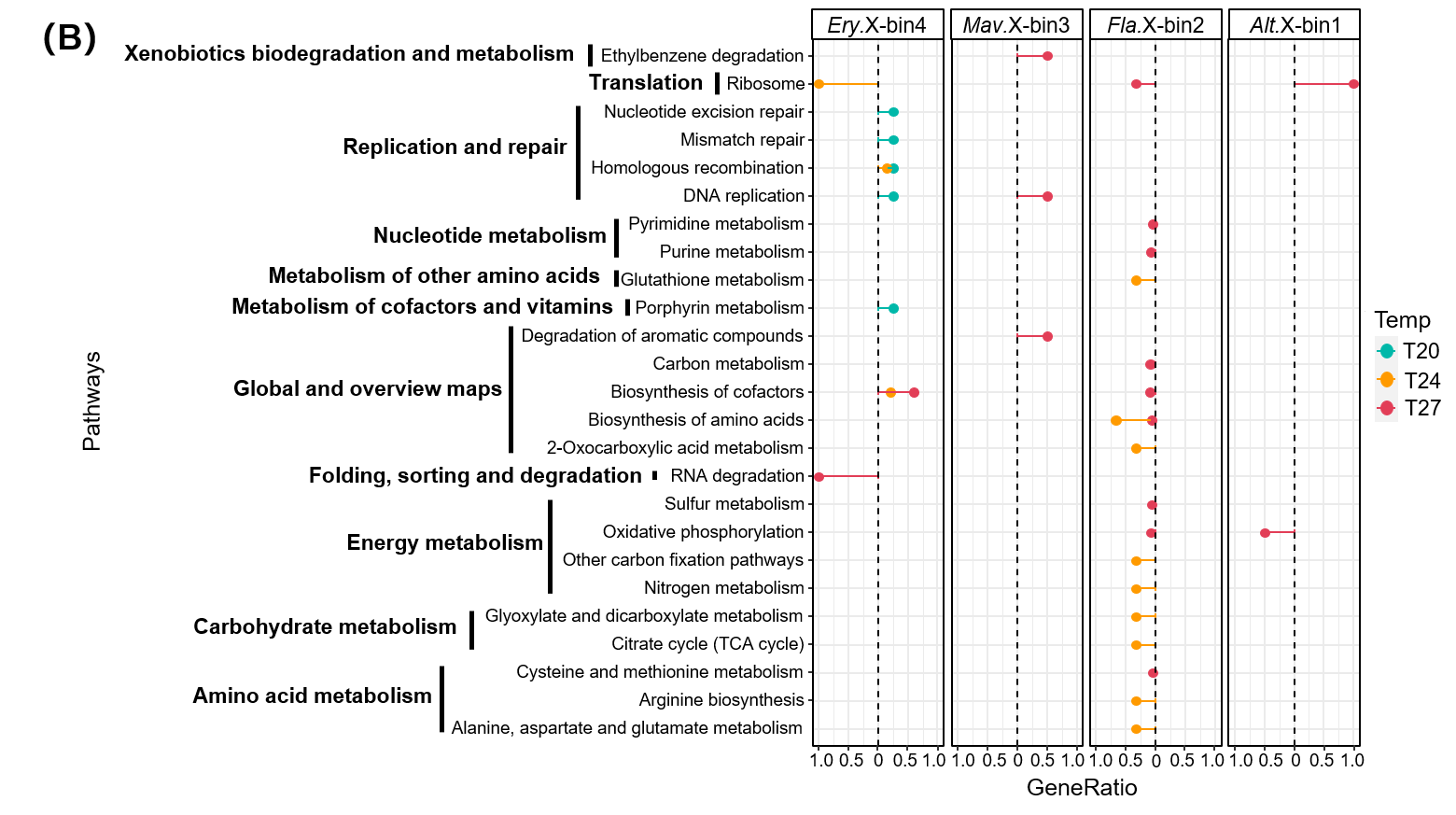


FIG S4 KEGG enrichment analysis of differentially expressed genes (DEGs) in binned heterotrophic bacteria from *Synechococcus* sp. YX04-1 (A) and XM-24 (B) co-cultures. The x-axis shows GeneRatio, and the y-axis lists enriched pathways. Branches to the left and right of the zero x-axis indicate significantly enriched down- and up-regulated pathways (*p.*adj < 0.05) under iron limitation at different temperatures. Temperatures are represented by cyan (T20), yellow (T24), and red (T27). Abbreviations: Temp: Temperature; *Bal.*Y-bin1: *Balneola* sp. Y-bin1; *Mar*.Y-bin2: *Marinobacter* sp. Y-bin2; *Rhi*.Y-bin3: *Rhizobium* sp. Y-bin3; *Rhi*.Y-bin4: *Rhizobiaceae* sp. Y-bin4; *Mam*.Y-bin5: *Mameliella* sp. Y-bin5; *Phy*.Y-bin6: *Phycisphaeraceae* sp. Y-bin6; *Alt*.X-bin1: *Alteromonas* sp. X-bin1; *Fla*.X-bin2: *Flavobacteriaceae* sp. X-bin2; *Mav*.X-bin3: *Marivita* sp. X-bin3; *Ery*.X-bin4: *Erythrobacter* sp. X-bin4.


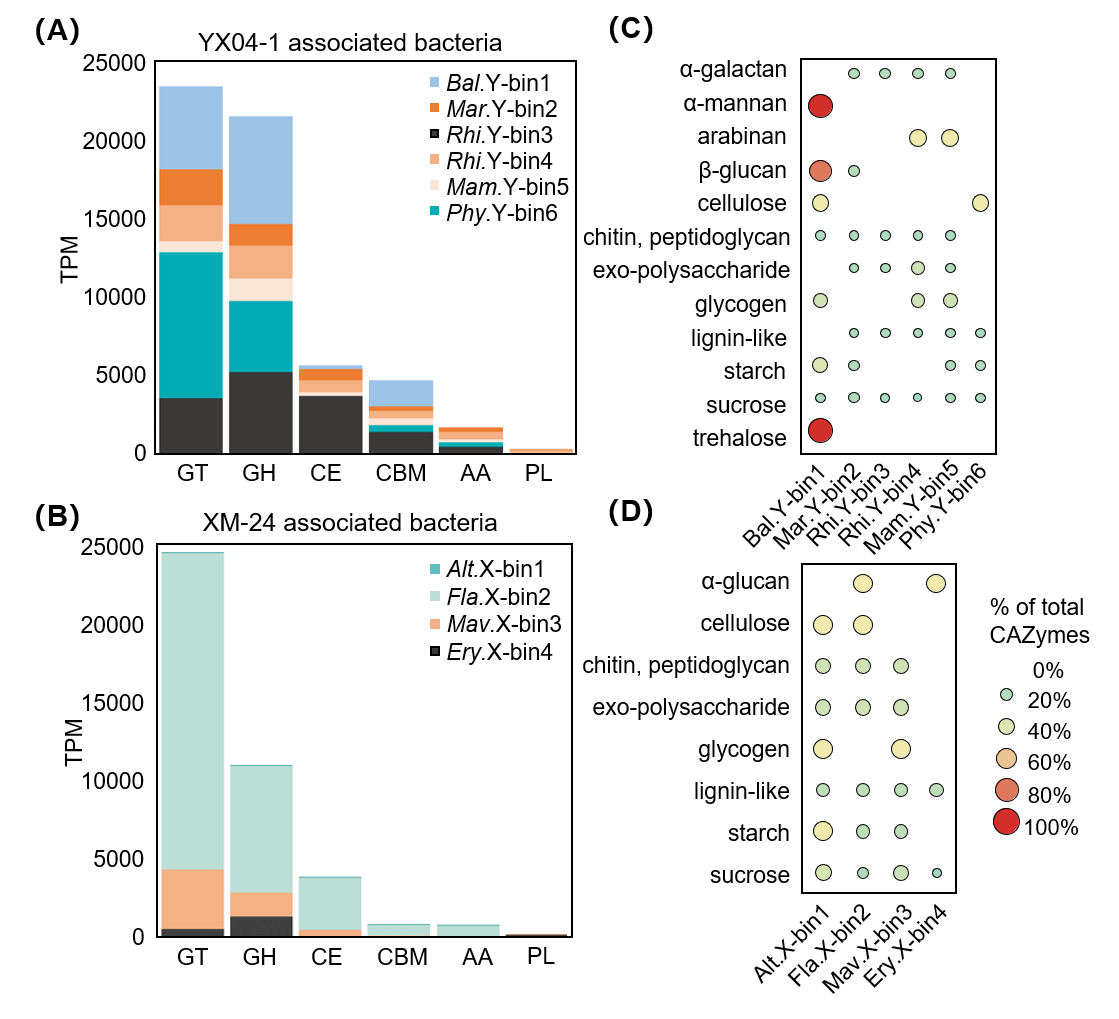


FIG S5 Stacked bar plots show CAZyme expression (in TPM) by category for binned heterotrophic bacteria in *Synechococcus* sp. YX04-1 (A) and XM-24 (B) co-cultures. Bubble plots indicate the distribution of target substrate-degrading CAZymes across different bins in *Synechococcus* sp. YX04-1 (C) and XM-24 (D) co-cultures. Bubble size and color represent the proportion of CAZymes in one bin relative to the total CAZymes in all heterotrophic bacteria across various substrates. Abbreviations: GH: glycoside hydrolases, CE: carbohydrate esterases, AA: enzymes for auxiliary activities, CBM: carbohydrate-binding module, PL: polysaccharide lyases. *Bal.*Y-bin1: *Balneola* sp. Y-bin1; *Mar*.Y-bin2: *Marinobacter* sp. Y-bin2; *Rhi*.Y-bin3: *Rhizobium* sp. Y-bin3; *Rhi*.Y-bin4: *Rhizobiaceae* sp. Y-bin4; *Mam*.Y-bin5: *Mameliella* sp. Y-bin5; *Phy*.Y-bin6: *Phycisphaeraceae* sp. Y-bin6; *Alt*.X-bin1: *Alteromonas* sp. X-bin1; *Fla*.X-bin2: *Flavobacteriaceae* sp. X-bin2; *Mav*.X-bin3: *Marivita* sp. X-bin3; *Ery*.X-bin4: *Erythrobacter* sp. X-bin4.


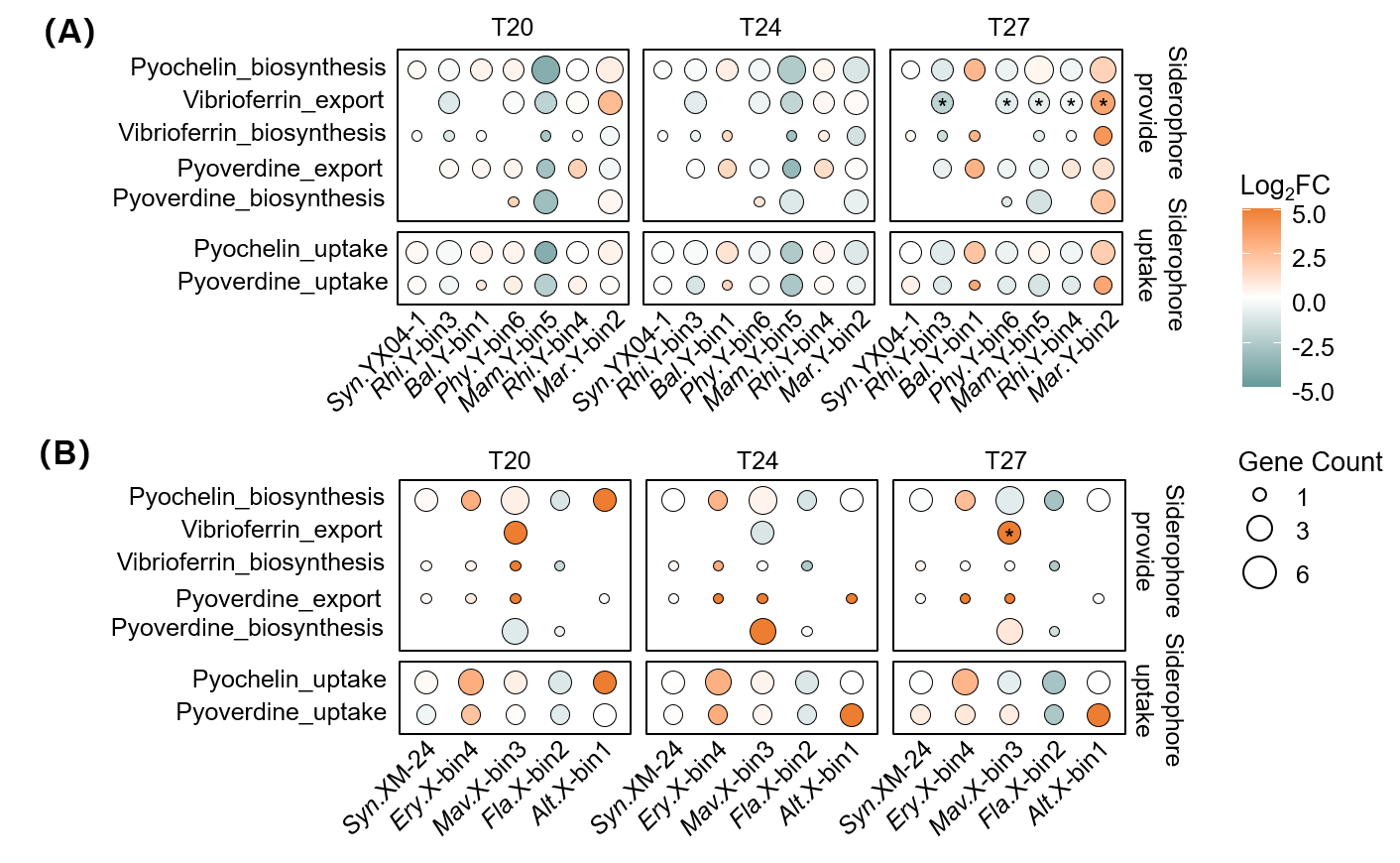


FIG S6 Heatmaps show changes in gene expression patterns of siderophore-related pathways in binned bacteria from *Synechococcus* sp. YX04-1 (A) and XM-24 (B) co-cultures under iron limitation at T20, T24, and T27. Asterisks denote significantly changed pathways (|log_2_FC| > 1 and *p < 0.05*). Bubble size represents the number of genes matching the corresponding function. Orange and cyan represent upregulated and downregulated pathways, respectively. Abbreviations: *Syn*.YX04-1: *Synechococcus* sp. YX04-1; *Bal.*Y-bin1: *Balneola* sp. Y-bin1; *Mar*.Y-bin2: *Marinobacter* sp. Y-bin2; *Rhi*.Y-bin3: *Rhizobium* sp. Y-bin3; *Rhi*.Y-bin4: *Rhizobiaceae* sp. Y-bin4; *Mam*.Y-bin5: *Mameliella* sp. Y-bin5; *Phy*.Y-bin6: *Phycisphaeraceae* sp. Y-bin6; *Syn*.XM-24: *Synechococcus* sp. XM-24; *Alt*.X-bin1: *Alteromonas* sp. X-bin1; *Fla*.X-bin2: *Flavobacteriaceae* sp. X-bin2; *Mav*.X-bin3: *Marivita* sp. X-bin3; *Ery*.X-bin4: *Erythrobacter* sp. X-bin4.


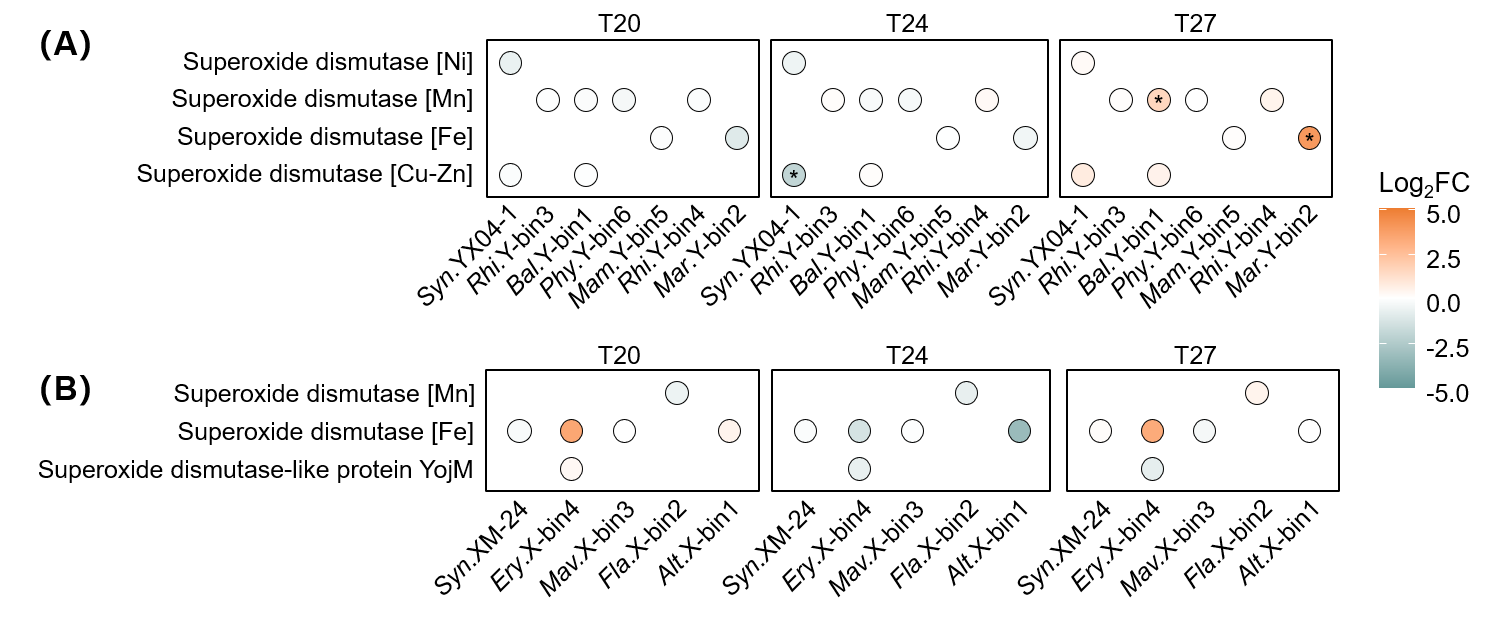


FIG S7 Heatmaps show changes in SOD-related gene expression patterns under iron limitation in binned bacteria from *Synechococcus* sp. YX04-1 (A) and XM-24 (B) co-cultures at T20, T24, and T27. Asterisks denote significantly changed genes (|log_2_FC| > 1 and *p*.adj *< 0.05*). Orange and cyan represent upregulated and downregulated genes, respectively. Abbreviations: *Syn*.YX04-1: *Synechococcus* sp. YX04-1; *Bal.*Y-bin1: *Balneola* sp. Y-bin1; *Mar*.Y-bin2: *Marinobacter* sp. Y-bin2; *Rhi*.Y-bin3: *Rhizobium* sp. Y-bin3; *Rhi*.Y-bin4: *Rhizobiaceae* sp. Y-bin4; *Mam*.Y-bin5: *Mameliella* sp. Y-bin5; *Phy*.Y-bin6: *Phycisphaeraceae* sp. Y-bin6; *Syn*.XM-24: *Synechococcus* sp. XM-24; *Alt*.X-bin1: *Alteromonas* sp. X-bin1; *Fla*.X-bin2: *Flavobacteriaceae* sp. X-bin2; *Mav*.X-bin3: *Marivita* sp. X-bin3; *Ery*.X-bin4: *Erythrobacter* sp. X-bin4.
